# Supplementary material for: Teenage mothers report poor health and economic functioning in Western Kenya: A call to action
Source: PLOS Glob Public Health. 2025 Jul 15;5(7):e0004732. doi: 10.1371/journal.pgph.0004732 (PMC12262858; doi:10.1371/journal.pgph.0004732)
Supplement: S1 Appendix — Fig A. Participant flow chart showing the original sample of LHS respondents that includes both men and women (N = 7,250) and how each study eligibility criteria affected sample size. Table A. Regression results for education and health outcomes presented in table format. Table B. Regression results for mental health outcomes presented in table format. Table C. Regression models using alternative definitions of child mortality as the outcome variable. Table D. Sensitivity analysis: logistic regression models with marginal effects presented in table. Table E. Sensitivity analysis: probit regression models with marginal effects presented in table. Table F. Sensitivity analysis: regression model excluding older participants (born before 1980s). Table G. Sensitivity analysis: regression model restricting sample to participants with non-missing data for each outcome variable. (PDF) [file pgph.0004732.s001.pdf]

Teen Mothers Report Poor Health and Economic Functioning in Western Kenya: A Call to Action

Aleksandra Jakubowski, Elizabeth Nakiyingi, Jane Wamae, Samuel Oyugi, Joseph R. Starnes, Sandra Mudhune, Benson Nyawade, Willys Ochieng, Erick Kelvin, Tom Odhong, Ash Rogers, Richard Wamai

S1 Appendix

Table of Contents

Fig A. Participant flow chart showing the original sample of LHS respondents that includes both men and women (N=7,250) and how each study eligibility criteria affected sample size.* *2

Table A. Regression results for education and health outcomes presented in table format.* *3

Table B. Regression results for mental health outcomes presented in table format.* *3

Table C. Regression models using alternative definitions of child mortality as the outcome variable.* *4

Table D. Sensitivity analysis: logistic regression models with marginal effects presented in table.* *4

Table E. Sensitivity analysis: probit regression models with marginal effects presented in table.* *5

Table F. Sensitivity analysis: regression model excluding older participants (born before 1980s).* *5

Table G. Sensitivity analysis: regression model restricting sample to participants with non-missing data for each outcome variable.* *6

Fig A. Participant flow chart showing the original sample of LHS respondents that includes both men and women (N=7,250) and how each study eligibility criteria affected sample size.

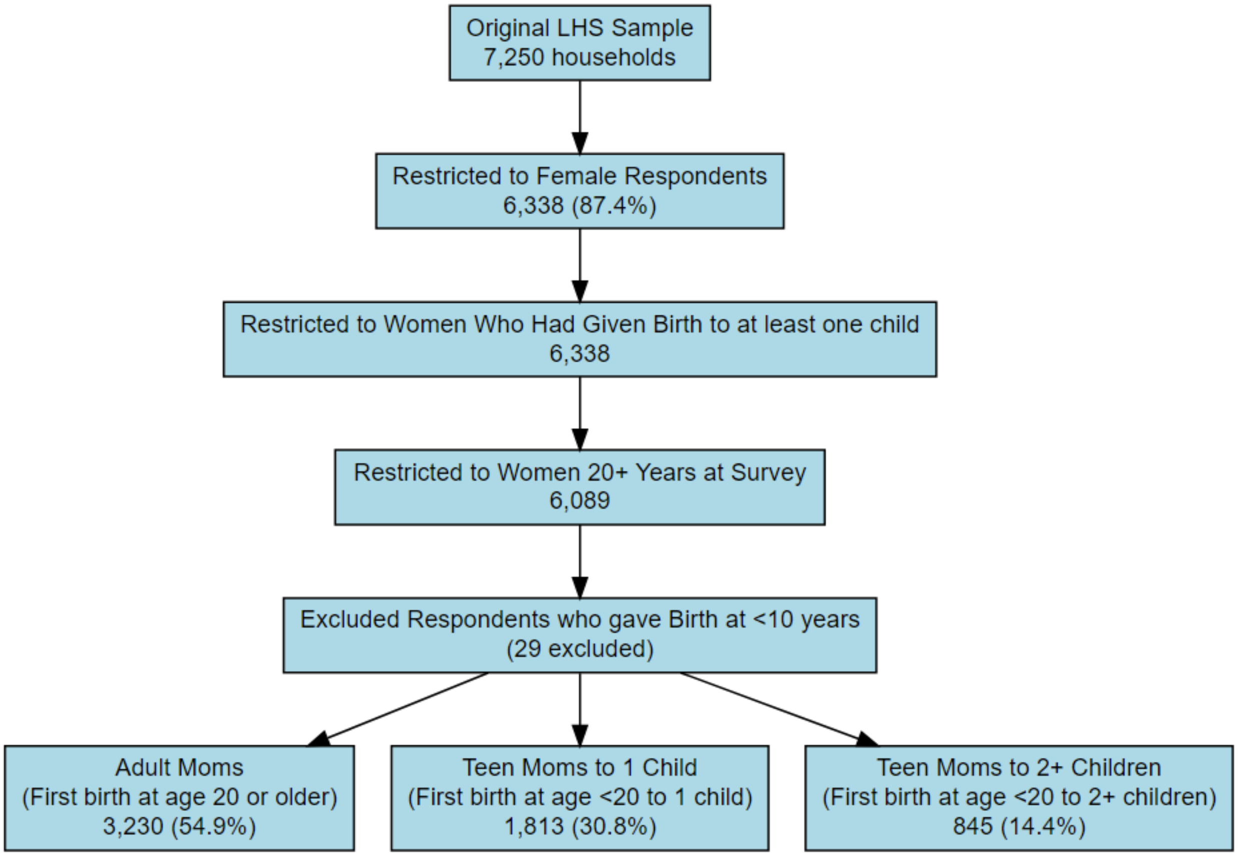

Table A. Regression results for education and health outcomes presented in table format.

	Completed primary education or more	Has loans	Went to sleep hungry last 4 weeks	Any children died	Short birth spacing	Poorest mental health scores
Teen mom, 1 child	-12.2***	4.3***	3.1**	5.6***	-3.1*	4.2**
[95% CI]	[-14.9, -9.4]	[1.8, 6.8]	[1.2, 5.0]	[4.1, 7.0]	[-5.5, -0.7]	[1.3, 7.1]
Teen mom, 2+ children	-27.6***	7.7***	9.2***	11.9***	24.9***	9.2***
[95% CI]	[-31.4, -23.8]	[4.2, 11.2]	[6.6, 11.8]	[9.9, 13.9]	[21.6, 28.2]	[5.2, 13.2]
Control mean	53.9%	18.6%	7.7%	3.4%	27.9%	28.2%
Observations	5,681	5,536	5,679	5,681	5,681	5,681
Wald test	<0.001	0.052	<0.001	<0.001	<0.001	0.012

Notes: Results from adjusted regression models. Each column represents a separate regression model adjusted for age, age-squared, marital status, religion, household size, wealth index, region of residence, and birth cohort fixed effect. Coefficients are interpreted as percentage point change from the control mean (adult women). 95% CIs presented in brackets. P-value notation: *** p<.001, ** p<.01, * p<.05

Table B. Regression results for mental health outcomes presented in table format.

	Little interest in doing things	Feeling down, depressed, or hopeless?	Trouble falling or staying asleep	Feeling tired or having little energy	Poor appetite or overeating	Feeling bad about yourself	Trouble concentrating	Moving or speaking slowly
Teen mom, 1 child	7.9**	6.6*	4.5	6.9*	2.5	6.9**	1.6	7.0**
[95% CI]	[2.6, 13.2]	[1.4, 11.8]	[-0.7, 9.7]	[1.6, 12.3]	[-2.6, 7.6]	[1.9, 11.9]	[-3.2, 6.4]	[2.2, 11.8]
Teen mom, 2+ children	12.1**	10.7**	13.2***	13.1***	13.9***	13.5***	10.6**	11.8***
[95% CI]	[4.8, 19.4]	[3.5, 17.8]	[6.0, 20.4]	[5.7, 20.5]	[6.9, 21.0]	[6.6, 20.4]	[3.9, 17.2]	[5.2, 18.5]
Control mean	61.8%	63.3%	62.6%	65.7%	56.2%	52.5%	46.2%	42.4%
Observations	5,663	5,674	5,659	5,670	5,661	5,644	5,645	5,623
Wald test	0.249	0.256	0.0161	0.0945	0.00118	0.0558	0.00713	0.147

Notes: Results from adjusted regression models. Each column represents a separate regression model adjusted for age, age-squared, marital status, religion, household size, wealth index, region of residence, and birth cohort fixed effect. Coefficients are interpreted as percentage point change from the control mean (adult women). 95% CIs presented in brackets. P-value notation: *** p<.001, ** p<.01, * p<.05

Table C. Regression models using alternative definitions of child mortality as the outcome variable.

	Any child died	Any child <5 died	Any infant died
Teen mom, 1 child	5.6***	0.7*	0.4
[95% CI]	[4.1, 7.0]	[0.1, 1.2]	[-0.1, 0.9]
Teen mom, 2+ children	11.9***	1.3***	0.5
[95% CI]	[9.9, 13.9]	[0.6, 2.1]	[-0.1, 1.2]
Control mean (adult mom)	3.4%	0.68%	0.49%
	5,681	5,681	5,681

Table D. Sensitivity analysis: logistic regression models with marginal effects presented in table.

	Completed primary education or more	Has loans	Went to sleep hungry last 4 weeks	Any children died	Short birth spacing	Poorest mental health scores
Teen mom, 1 child	-12.0***	4.5***	3.6***	5.3***	-3.3**	4.2**
[95% CI]	[-14.8, -9.2]	[1.9, 7.0]	[1.7, 5.4]	[3.7, 6.8]	[-5.7, -0.9]	[1.4, 7.1]
Teen mom, 2+ children	-28.0***	7.8***	8.6***	11.3***	22.5***	9.0***
[95% CI]	[-31.8, -24.3]	[4.2, 11.4]	[5.9, 11.4]	[8.6, 14.0]	[18.6, 26.5]	[5.0, 13.1]
Control mean	53.9%	18.6%	7.7%	3.4%	27.9%	28.2%
Observations	5,681	5,536	5,679	5,681	5,681	5,681

Notes: Marginal effects calculated after logit models. Each column represents a separate regression model adjusted for age, age-squared, marital status, religion, household size, wealth index, region of residence, and birth cohort fixed effect. P-value notation: *** p<.001, ** p<.01, * p<.05

Table E. Sensitivity analysis: probit regression models with marginal effects presented in table.

	Completed primary education or more	Has loans	Went to sleep hungry last 4 weeks	Any children died	Short birth spacing	Poorest mental health scores
Teen mom, 1 child	-11.9***	4.5***	3.4***	5.2***	-2.8*	4.2**
[95% CI]	[-14.7, -9.1]	[2.0, 7.0]	[1.6, 5.2]	[3.7, 6.7]	[-5.1, -0.4]	[1.3, 7.1]
Teen mom, 2+ children	-27.6***	8.0***	8.8***	11.3***	23.4***	9.0***
[95% CI]	[-31.3, -23.9]	[4.4, 11.6]	[6.0, 11.6]	[8.6, 14.0]	[19.6, 27.3]	[4.9, 13.1]
Control mean	53.9%	18.6%	7.7%	3.4%	27.9%	28.2%
Observations	5,681	5,536	5,679	5,681	5,681	5,681

Notes: Marginal effects calculated after probit models. Each column represents a separate regression model adjusted for age, age-squared, marital status, religion, household size, wealth index, region of residence, and birth cohort fixed effect. P-value notation: *** p<.001, ** p<.01, * p<.05

Table F. Sensitivity analysis: regression model excluding older participants (born before 1980s).

	Completed primary education or more	Has loans	Went to sleep hungry last 4 weeks	Any children died	Short birth spacing	Poorest mental health scores
Teen mom, 1 child	-12.8***	5.3***	3.2***	4.6***	-4.3***	5.8***
[95% CI]	[-15.7, -9.8]	[2.7, 8.0]	[1.3, 5.1]	[3.2, 6.0]	[-6.8, -1.8]	[2.8, 8.8]
Teen mom, 2+ children	-29.1***	9.0***	9.2***	10.2***	21.1***	11.2***
[95% CI]	[-33.3, -24.9]	[5.2, 12.8]	[6.5, 11.9]	[8.2, 12.2]	[17.6, 24.6]	[6.9, 15.6]
Control mean	58.7%	17.1%	6.1%	2.7%	24.1%	26.9%
Observations	5,200	5,057	5,198	5,200	5,200	5,200

Notes: Results from adjusted regression models that excludes the eldest participants, those born before 1980s. Each column represents a separate regression model adjusted for age, age-squared, marital status, religion, household size, wealth index, region of residence, and birth cohort fixed effect. Coefficients are interpreted as percentage point change from the control mean (adult women). 95% CIs presented in brackets. P-value notation: *** p<.001, ** p<.01, * p<.05

Table G. Sensitivity analysis: regression model restricting sample to participants with non-missing data for each outcome variable**.**

	Completed primary education or more	Has loans	Went to sleep hungry last 4 weeks	Any children died	Short birth spacing	Poorest mental health scores
Teen mom, 1 child	-12.4***	4.3***	3.2***	5.7***	-3.4**	4.3**
[95% CI]	[-15.2, -9.6]	[1.8, 6.9]	[1.3, 5.1]	[4.2, 7.2]	[-5.8, -0.9]	[1.3, 7.2]
Teen mom, 2+ children	-27.6***	7.7***	9.3***	12.1***	24.9***	8.9***
[95% CI]	[-31.5, -23.8]	[4.2, 11.2]	[6.7, 11.9]	[10.0, 14.1]	[21.5, 28.2]	[4.8, 12.9]
Control mean	53.9%	18.6%	7.8%	3.4%	28.4%	28.9%
Observations	5,534	5,534	5,534	5,534	5,534	5,534

Notes: Results from adjusted regression models that restricts the analytic sample to participants who have nonmissing data on all variables. Each column represents a separate regression model adjusted for age, age-squared, marital status, religion, household size, wealth index, region of residence, and birth cohort fixed effect. Coefficients are interpreted as percentage point change from the control mean (adult women). 95% CIs presented in brackets. P-value notation: *** p<.001, ** p<.01, * p<.05
