# Supplementary material for: Teenage mothers report poor health and economic functioning in Western Kenya: A call to action
Source: PLOS Glob Public Health. 2025 Jul 15;5(7):e0004732. doi: 10.1371/journal.pgph.0004732 (PMC12262858; doi:10.1371/journal.pgph.0004732)
Supplement: Attachment — Submitted filename: Jakubowski et al - PLOS GH - rebuttal.pdf [file pgph.0004732.s003.pdf]

Northeastern University

Aleksandra Jakubowski PhD MPH
Assistant Professor
Department of Public Health and Health Sciences
Department of Economics
Northeastern University
360 Huntington Ave, Robinson 408A
Boston, MA 02115

May 16, 2025

Dear Dr. Ranganathan:

Thank you for the opportunity to submit a revised version of our manuscript, "Teen Mothers Report Poor Health and Economic Functioning in Western Kenya: A Call to Action," to *PLOS Global Public Health*. We appreciate the editors' and reviewers' openness to our analyses, and we found the comments constructively challenging throughout. We reproduced the comments verbatim below, with our responses.

The Reviewers raised important feedback about the need to strengthen the writing of the manuscript, clarify methodological details, and elaborate on the contextual relevance and implications of our findings. We have revised the manuscript accordingly, and we believe these changes have significantly improved the quality and clarity of the paper.

If there is any additional information we can provide or further revisions we can make, please do not hesitate to let us know. Thank you for your consideration and interest in this work.

Sincerely,

Aleksandra Jakubowski on behalf of study authors

-------------------------- Journal Editor’s comments --------------------------

1. Please include a complete copy of PLOS’ questionnaire on inclusivity in global research in your revised manuscript. Our policy for research in this area aims to improve transparency in the reporting of research performed outside of researchers’ own country or community. The policy applies to researchers who have travelled to a different country to conduct research, research with Indigenous populations or their lands, and research on cultural artefacts. The questionnaire can also be requested at the journal’s discretion for any other submissions, even if these conditions are not met. Please find more information on the policy and a link to download a blank copy of the questionnaire here: https://journals.plos.org/globalpublichealth/s/best-practices-in-research-reporting. Please upload a completed version of your questionnaire as Supporting Information when you resubmit your manuscript.

Response: We have completed the PLOS Inclusivity in Global Research questionnaire as requested and have uploaded it as a Supporting Information file in our revised submission.

2. Please insert an Ethics Statement at the beginning of your Methods section, under a subheading 'Ethics Statement'.

Response: We updated the heading of the Ethics section to “Ethics Statement” and we moved it to the beginning of the Methods section.

3. Please provide separate figure files in .tif or .eps format.

   For more information about figure files please see our guidelines:

   https://journals.plos.org/globalpublichealth/s/figures
   https://journals.plos.org/globalpublichealth/s/figures#loc-file-requirements

Response: We saved each figure as .tiff file and uploaded these through the submission portal.

4. We have noticed that you have cited Table 3 in the manuscript file but there are no corresponding tables in the manuscript. Please amend your manuscript to include this table, noting that tables should not be uploaded as individual files.

Response: Thank you for pointing this out. We were using an older version of numbering the supplement tables. We have now updated the note for Fig 2 to remove the reference to Supplement Table 3 and we instead refer to it as “S1 Table A” (pg. 11). We also reviewed how we refer to all other supplement tables and figures and made a change throughout (see legends for Fig 3 and 4, pg 10-11).

5. We have noticed that you have uploaded Supporting Information files, but you have not included a list of legends. Please add a full list of legends for your Supporting Information files after the references list.

Response: We have now included a complete list of legends for all Supporting Information files at the end of the manuscript, following the references section, as requested (pg. 19-20).

6. In the online submission form, you indicated that “De-identified data will be made available upon request.”.

All PLOS journals now require all data underlying the findings described in their manuscript to be freely available to other researchers, either

- In a public repository,
- Within the manuscript itself, or
- Uploaded as supplementary information.

This policy applies to all data except where public deposition would breach compliance with the protocol approved by your research ethics board. If your data cannot be made publicly available for ethical or legal reasons (e.g., public availability would compromise patient privacy), please explain your reasons by return email and your exemption request will be escalated to the editor for approval. Your exemption request will be handled independently and will not hold up the peer review process, but will need to be resolved should your manuscript be accepted for publication. One of the Editorial team will then be in touch if there are any issues.

Response: Thank you for the clarification regarding PLOS's data availability policy. We confirm that the de-identified data used in this study will be made publicly available, specifically, we will upload the dataset to the Harvard Repository, where it will be accessible to other researchers upon publication.

7. Some material included in your submission may be copyrighted. According to PLOS's copyright policy, authors who use figures or other material (e.g., graphics, clipart, maps) from another author or copyright holder must demonstrate or obtain permission to publish this material under the Creative Commons Attribution 4.0 International (CC BY 4.0) License used by PLOS journals. Please closely review the details of PLOS's copyright requirements here: PLOS Licenses and Copyright. If you need to request permissions from a copyright holder, you may use PLOS's Copyright Content Permission form.

   Please respond directly to this email or email the journal office and provide any known details concerning your material's license terms and permissions required for reuse, even if you have not yet obtained copyright permissions or are unsure of your material's copyright compatibility.

   Potential Copyright Issues:

   a. Figure 1: please (a) provide a direct link to the base layer of the map (i.e., the country or region border shape) and ensure this is also included in the figure legend; and (b) provide a link to the terms of use / license information for the base layer image or shapefile. We cannot publish proprietary or copyrighted maps (e.g. Google Maps, Mapquest) and the terms of use for your map base layer must be compatible with our CC-BY 4.0 license.

   Note: if you created the map in a software program like R or ArcGIS, please locate and indicate the source of the basemap shapefile onto which data has been plotted.

   If your map was obtained from a copyrighted source please amend the figure so that the base map used is from an openly available source. Alternatively, please provide explicit written permission from the copyright holder granting you the right to publish the material under our CC-BY 4.0 license.

   Please note that the following CC BY licenses are compatible with PLOS license: CC BY 4.0, CC BY 2.0 and CC BY 3.0, meanwhile such licenses as CC BY-ND 3.0 and others are not compatible due to additional restrictions.

   If you are unsure whether you can use a map or not, please do reach out and we will be able to help you. The following websites are good examples of where you can source open access or public domain maps:

- U.S. Geological Survey (USGS) - All maps are in the public domain. (http://www.usgs.gov)
- PlaniGlobe - All maps are published under a Creative Commons license so please cite "PlaniGlobe, http://www.planiglobe.com, CC BY 2.0" in the image credit after the caption. (http://www.planiglobe.com/?lang=enl)
- Natural Earth - All maps are public domain. (http://www.naturalearthdata.com/about/terms-of-use/)

Response: We updated the map and have added the following statement about Figure 1 to the legend (pg. 5): "The map in Fig 1 was created in QGIS using basemap shapefiles available through the Database of Global Administrative Areas. These data are freely available for use in academic publishing (https://gadm.org/license.html). The figure was previously used in other PLOS publications (https://journals.plos.org/plosone/article?id=10.1371/journal.pone.0256555)."

8. Please review your reference list to ensure that it is complete and correct. If you have cited papers that have been retracted, please include the rationale for doing so in the manuscript text, or remove these references and replace them with relevant current references. Any changes to the reference list should be mentioned in the rebuttal letter that accompanies your revised manuscript. If you need to cite a retracted article, indicate the article's retracted status in the References list and also include a citation and full reference for the retraction notice.

Response: We reviewed the references in detail and the following references were edited/added:

1. World Health Organization. *Adolescent pregnancy: Issues in adolescent health and development (WHO Discussion Papers on Adolescence).* 2004.
2. Achia TN, Mageto L, Kironji A. Spatial variation and risk factors of teenage pregnancy in Kenya. *PLoS One.*2023;18(4):e0283943.
3. Bozkurt P, Özcan NK. Comparison of adolescent and adult mothers in terms of maternal and neonatal outcomes: A cross-sectional study. *Perspectives in Psychiatric Care.* 2021;57(2):788–95.
4. Flor LS, Reitsma MB, Gupta V, Ng M, Gakidou E. The effect of women's access to education on maternal health in sub-Saharan Africa: a secondary analysis of existing data. *Lancet Global Health.* 2022;10(8):e1111–e1122.
5. Godha D, Hotchkiss DR, Gage AJ. Association between child marriage and reproductive health outcomes and service utilization: A multi-country study from South Asia. *Journal of Adolescent Health.* 2013;52(5):552–8.
6. Govender D, Naidoo S, Taylor M. Scoping review of risk factors of and interventions for adolescent repeat pregnancies: a public health perspective. *African Journal of Primary Health Care & Family Medicine.* 2020;12(1).
7. Hindin MJ, Fatusi AO. Adolescent sexual and reproductive health in developing countries: An overview of trends and interventions. *International Perspectives on Sexual and Reproductive Health.* 2009;35(2):58–62.
8. Khairani CD, Andriani D, Suherman S. The association between knowledge, attitude and adolescent pregnancy in Indonesia. *Malaysian Journal of Medicine and Health Sciences.* 2022;18(SUPP16):139–44.
9. Koenig MA, Zablotska I, Lutalo T, Nalugoda F, Wagman J, Gray R. Coerced first intercourse and reproductive health among adolescent women in Rakai, Uganda. *International Family Planning Perspectives.* 2004;30(4):156–63.
10. Lawan UM, Takai IU, Ishaq H. Sociocultural factors influencing decision-making related to fertility among the Kanuri tribe of north-eastern Nigeria. *Annals of African Medicine.* 2013;12(3):160–4.
11. Loaiza E, Liang M. Adolescent pregnancy: A review of the evidence. *UNFPA Technical Report.* 2013.

12. Miller E, Decker MR, Reed E, Raj A, Hathaway JE, Silverman JG. Male partner pregnancy-promoting behaviors and adolescent partner violence: Findings from a qualitative study with adolescent females. *Ambulatory Pediatrics.* 2007;7(5):360–6.
13. Moussaoui D, Nofal A. Mental health and psychosocial consequences of child marriage. *Journal of Child and Adolescent Mental Health.* 2021;33(1):53–62.
14. Neal S, Matthews Z, Frost M, Fogstad H, Camacho AV, Laski L. Childbearing in adolescents aged 12–15 years in low resource countries: a neglected issue. *New estimates from demographic and household surveys. Acta Obstetricia et Gynecologica Scandinavica.* 2012;91(9):1114–8.
15. Nguyen MC, Wodon Q. Impact of child marriage on literacy and education attainment in Africa. *World Bank Policy Research Working Paper.* 2015;(WPS7589).
16. Nour NM. Health consequences of child marriage in Africa. *Emerging Infectious Diseases.* 2006;12(11):1644–9.
17. Okonofua F, Odemwingie R, Odimegwu C, Ajabor H, Adewuyi A. Men's and women's perceptions of the use of family planning and maternal health services in Ekpoma, Nigeria. *African Journal of Reproductive Health.*2013;17(3):94–105.
18. Raj A, McDougal L. Sexual violence and rape in India. *Lancet.* 2014;383(9920):865.
19. Raj A, Saggurti N, Balaiah D, Silverman JG. Prevalence of child marriage and its effect on fertility and fertility-control outcomes of young women in India: a cross-sectional, observational study. *Lancet.* 2009;373(9678):1883–9.
20. Rasanathan JJ, Damji N, Atsbeha T, Brune DR, Davis T, Dora C, et al. Ensuring multisectoral action on adolescent health and well-being: a review of successful country cases. *BMJ.* 2021;372:n385.
21. Santhya KG, Jejeebhoy SJ. Sexual and reproductive health of young people in India: A review of policies, laws and programmes. *Population Council Report.* 2012.
22. Temin M, Levine R. Start with a girl: A new agenda for global health. *Center for Global Development.* 2009.
23. Wado YD, Afework MF, Hindin MJ. Unintended pregnancies and the use of maternal health services in southwestern Ethiopia. *BMC International Health and Human Rights.* 2013;13(1):36.
24. Walker JA. Early marriage in Africa–Trends, harmful effects and interventions. *African Journal of Reproductive Health.* 2012;16(2):231–40.
25. Yaya S, Odusina EK, Uthman OA, Bishwajit G. What factors determine adolescent reproductive health service utilization in sub-Saharan Africa? Evidence from Benin, Mali and Nigeria. *BMC Public Health.* 2019;19(1):1–9.

------------------------- Reviewer 1 comments ----------------------------

Reviewer #1: Thank you so much for the opportunity to review this very meaningful paper, studying a previously underexplored area. While the content is incredibly insightful the scientific writing of this paper could be very much improved.

Response: Thank you for your constructive feedback. We detail how we address each of your comments below.

1. Overall:
Please correct grammatical errors throughout the piece, some have been pointed others have not. Would strongly suggest reading through the whole piece and improving writing, grammar and sentence structure to improve readability - especially in the introduction and discussion

Response: Thank you for your careful read of the manuscript and providing examples of where language can be improved. We provide details on how we responded to each specific comment below. We also reviewed the paper carefully again to ensure there were no errors in the manuscript.

2. Abstract:
   1. 'Teenage pregnancy remains a critical issue in Kenya, with 15% of girls 15-19 having been pregnant.' Please fix the grammar of this sentence, e.g., 'Teenage pregnancy remains a critical issue in Kenya, with 15% of girls aged 15-19 having been pregnant.

Response: Thank you for this suggestion. We updated the sentence, and it now reads as follows (pg. 2 line 1):

> "Teenage pregnancy remains a critical issue in Kenya, with 15% of girls aged 15-19 having been pregnant"

3. Intro:
   The paper might benefit with a clear statement upfront defining teen pregnancy. E.g. Teen pregnancy is defined as pregnancy from the ages of 15-19… (ref) This is defined in the methods but as is it is the premise of the paper an upfront definition is always helpful.

Response: Great suggestion, thank you. We added the WHO definition of teen pregnancy and childbirth to the first sentence and added a citation to a WHO document in which teenage pregnancy is defined to the Introduction (Page 3; line 2 and 3). The revised text now states:

> "Teenage pregnancy and childbearing (defined by the World Health Organization (WHO) as a pregnancy or birth occurring to a woman aged 10-19)(1) remains a significant public health problem globally and is highest in sub-Saharan Africa(SSA)(2)."

4. In 2022, Migori County (the focus of this study) had the fifth highest teen pregnancy in Kenya at 23% of women 15-19 years and adult (15-49) HIV prevalence of 9.73%.
   Please clarify ages counted as adult for HIV prevalence? A teenage pregnancy is 15-19 years of age however adult HIV prevalence counts as 15 years old or onwards?

Response: Thank you for this helpful comment. We are using the official Kenyan national statistics on HIV and are citing the adult HIV prevalence according to the statistics cited by the Ministry of Health. We checked other commonly used sources for HIV statistics in Kenya, such as the Kenya Demographic and Health Survey (KDHS) and the Kenya Population-based HIV Impact Assessment (KENPHIA), and all of these resources report adult HIV prevalence for age range of 15-49 or 15+. Accordingly, we are keeping the HIV prevalence statistics for the age group of 15-49 year olds, which is the most relevant age range for this study.

We acknowledge your point that individuals aged 15–17 may not be considered adults by some definitions. In response, we have removed the term "adult" from the text and now specify the exact age range (15–49 years) when referring to HIV prevalence.

As part of this revision, we also concluded that specifying HIV prevalence among women is more relevant to our study than reporting the overall population prevalence (men and women combined). We have updated the statistics accordingly, and the revised text now reads (pg. 3):

"In 2022, Migori County (the focus of this study) had the fifth highest teen pregnancy rate in Kenya at 23% among of girls aged 15-19 years and an HIV prevalence of 9.73% among individuals aged 15 –49 years."

5. Methods:
   These sampling and survey techniques have been extensively described in prior studies.(38) Briefly, we implemented the World Health Organization (WHO) Expanded Programme of Immunization (EPI) method(40) to randomly select the sampling frame, and oversampled households that included women with children under 5 years.(38)
   Would suggest flipping these sentences around giving a clear explanation of methodology for sampling and then mentioning extensive description elsewhere.

Response: Thank you for your comment. We have revised this paragraph to give a clear explanation of sampling methodology before mentioning extensive description elsewhere. The revised paragraph states (pg. 5):

"Located in the Lake Victoria region of southwestern Kenya, Migori County is a mostly rural area with a population of over 1 million people (41). Administratively, there are 10 sub-counties, each with multiple sub-divisions called wards (42). We randomly selected households from 8 wards using a hybrid sampling strategy adapted from the World Health Organization's Expanded Programme on Immunization (EPI). Each ward was divided into 127 grid squares using Geographic Information System (GIS) technology, with enumerators starting each day at a randomly generated central point within each grid. Households were then selected using the "spin-the-bottle" method, in which a random direction was chosen, and households were surveyed sequentially along that path(43). To reflect Lwala Community Alliance's programmatic focus on maternal and child health, households with children under five years of age were intentionally oversampled. Specifically, of the seven households surveyed within each grid square, at least five were required to include a child under five. Survey teams prioritized interviewing female heads of household when available, particularly due to modules on family planning and child health. Further details about sampling and survey techniques have been extensively described in prior publications (39)."

6. Would an eleven-year-old giving birth classify as a teen mother to a child? Is there a lower cut off age?

Response: Thank you for this important question. In our analysis, we defined teenage motherhood according to the World Health Organization's definition of a birth occurring to a woman aged 10-19 years old. According to this definition, an 11 year old child giving birth would classify as a teen mother.

The analytic sample excludes respondents who disclosed being younger than 10 years of age at the time they first became mothers to be consistent with the WHO definition of teen motherhood to minimize the risk of data quality issues such as recall bias. In total, 29 respondents were excluded due to this study restriction and we do not believe that their data would meaningfully affect the results.

We expanded on how we defined the study groups in the "Study groups" subsection of the Methods (pg 6):

"We defined teenage mothers according to the WHO definition as any birth that occurred to a woman ages 10 to 19 years old(2). We excluded respondents who reported giving birth before age 10 due to the potential for reporting errors and recall bias."

7. Is there a justification of the 25th percentile mental health cut off or is it arbitrary?

Response: Thank you for this comment. We used the top quartile as a cutoff to define poor mental health in order to identify women with the most severe depressive symptoms within our sample. This approach is consistent with practices in population-based studies where clinical diagnostic thresholds may be unavailable. Dividing data into quartiles is common in quantitative analysis and we used this specific cutoff to identify women whose mental health was most affected. We have added a brief justification in the methods section to clarify this on page 6:

> "We then calculated total mental health score for each participant and defined a poor mental health indicator as those women who scored in the top 25th percentile to identify those with the most severe symptoms within our sample in the absence of clinical diagnostic cutoffs."

8. 'The region fixed effect control for regional variation and birth cohort fixed effect accounted for temporal trends.' Fix grammar please.

Response: Thank you for pointing this out. We have corrected the grammar of the sentence, which now reads (pg7):

> "The region fixed effect controlled for regional variation, and the birth cohort fixed effect accounted for temporal trends."

9. The uncertainty surrounding child mortality alongside recall bias is not something that should be reported in the methodology but in the limitations section under discussion. What was conducted is what must be relayed in the methodology.

Response: Thank you for this suggestion. We believe that it is critical to mention the uncertainty surrounding child mortality data and recall bias in the Methods section because it helps explain the rationale for conducting specific sensitivity analyses or robustness checks. However, we agree that this information also belongs in the Discussion section under limitations and have ensured it is clearly stated there as well. The updated limitations section now states (pg. 16):

> "Our study had multiple limitations. The results can only be interpreted as associations given the cross-sectional nature of the data and the endogeneity of teen motherhood and economic functioning. We did not have sufficient sample size to estimate the association between teen motherhood and infant mortality with precision. The child mortality outcomes relied on self-reported birth and death histories, which may be subject to recall bias, especially for events that occurred many years prior to the survey. This may introduce uncertainty in distinguishing between infant and under-five mortality. The study used retrospective analysis techniques to assign women into study groups based on when they first became mothers. It is possible that recalling the years in which respondents' children were born includes some recall bias, but we do not expect this to affect our results. Finally, the rural, high HIV prevalence, and impoverished context of Migori County may limit the generalizability of results to wealthier, urban, or low HIV prevalence settings."

10. Does AMREF have a full form?

Response: Thank you for pointing out that we did not define this acronym. We now provide the full name of the IRB before stating the acronym. This clarification has been added to the Ethical Considerations section on page 4:

"The household survey's protocol and study design received approval from the African Medical and Research Foundation (AMREF) Health Africa Ethics and Scientific Review Committee on March 29, 2021 (AMREF-ESRC P452/2018)"

11. Results:
    '…1,813 (30.8%) Teen mothers' The T does not need to be capitalized.

Response: We have corrected this sentence so that "teen mothers" is no longer capitalized (pg 7). Thank you again for your detailed review of the paper.

12. For results reported in the text have n and % from the table in the text as well, e.g. 'Majority of the participants were married across all groups, and most were Seventh Day Adventist. (45.7%, n=1,457)' Repeat as needed where needed.

Response: The section the reviewer is referring to is the descriptive paragraph where we aim to briefly highlight a few key characteristics of the study participants. We believe that providing the n and % for each characteristic in this narrative section would detract from the main objective of the paper. These details are already fully presented in Table 1, and the purpose of the accompanying text is to guide the reader through the table and contextualize the statistics. To address the Reviewer's comment we limited the number of characteristics we provide in text and specify the % for each study group. The revised text now reads (pg. 8):

"Most study participants were married across all study groups (83.5% adult mothers, 89.5% teen mothers). Teen mothers to 2+ children lived in larger households compared to the other groups."

13. 'We found a clear wealth gradient among the three study groups: adult mothers were much more likely to live in the wealthiest households, and teen mothers to 2+ children were more concentrated in the least wealthy households. For example, 23.7% of adult mothers lived in the wealthiest households compared to 13.8% of teen mothers to 2+ children.'

    The second sentence is not an example of the first it is the verification of it, both sentences could be combined or at the very least the phrase for example could be removed.

Response: We have revised the text to be more concise and to illustrate the gradient. The revised text now states (Pg. 9):

"We found a clear wealth gradient among the three study groups: adult mothers were much more likely to live in the wealthiest households (23.7%), and teen mothers to 2+ children were more concentrated in the least wealthy households (22.7%). Most participants were born between 1980 and 1999."

14. Remove doubling and quadrupling.

Response: Although this is purely a stylistic revision, we updated the results section to remove the terms "doubling" and "quadrupling" and now present the exact percentages for each study group (pg. 9).

15. Keep terms like food insecurity, short birth spacing and child mortality consistent in the results (table, text and figures) as explained in the methods, no need to revert back to slept hungry or death of a child.

Response: We have revised the results section to ensure consistent terminology throughout the manuscript. Specifically, we now refer to the outcomes as food insecurity, child mortality, and short birth spacing, aligning with the definitions provided in the Methods section (pgs. 8-12).

16. Discussion:
Would not describe findings as 'striking' consistently.

Response: Thank you for your thorough review. We used this word twice in the manuscript and have removed the second instance to avoid repetition and ensure consistency in tone.

17. Perhaps elaborate on the reasons for short birth spacing? To identify areas of intervention?

Response: Thank you for this helpful suggestion. We have expanded the Discussion section to further explore possible reasons for short birth spacing among teen mothers, including limited access to postpartum family planning services, low reproductive autonomy, stigma from healthcare providers, and socio-cultural pressures. We have added supporting references for these explanations and emphasized the importance of adolescent-friendly reproductive health programs as critical areas for intervention. The revised text appears in the Discussion (pg. 12):

> "Among the study's most striking results, we found that women who had multiple children as teenagers were over three times more likely to experience the death of a child compared to those women who first gave birth as adults (38, 51). Extensive literature documents that adolescent mothers face increased risk of pregnancy and birth complications such as obstructed labor, preterm delivery, and postpartum hemorrhage(16-18). Our study identifies an additional factor contributing to higher child mortality rates among teen mothers: short birth spacing, which adversely affects both maternal and child health (52, 53). Women who had 2+ children as teens were 27pp more likely (over three times more likely in absolute terms) to have less than 2 years between their children, a well-established risk factor for child mortality(52, 53). This increased risk may stem from limited access to contraception and postpartum family planning counseling, stigma from healthcare providers, and societal pressures to have more children(54, 55). Teen mothers may also face barriers such as low autonomy in reproductive decision making and limited knowledge about birth spacing and contraception(56, 57). These findings underscore the urgent need for targeted postpartum family planning programs for teen mothers and adolescent friendly reproductive health services to address these barriers. (58)"

18. Instead of describing poor mental health what are the repercussions of the same?

Response: Great suggestion. In the revision, we have expanded the discussion to include the potential consequences of poor mental health among teen mothers. Specifically, we note that poor mental health can impair labor force participation worsening long-term economic outcomes, reduce caregiving capacity, and impact both maternal and child health outcomes (pg. 12-13):

> "Another key finding is the marked decline in mental health among teen mothers. Previous research has documented the isolation and stigma surrounding teen pregnancy(20, 30, 59, 60), factors that contribute to poor mental health outcomes. Our study documents that these mental health challenges persist into adulthood, underscoring the critical importance of addressing mental wellbeing of teen mothers. Among the eight PHQ-8 symptoms we measured, teen mothers to 2+ children scored

significantly higher on each item, including having little interest in things, feeling bad about oneself or like a failure, having trouble sleeping, concentrating, and feeling tired. The severity of poor health and socio-economic outcomes we observe likely contribute to this mental health burden. Our findings are in line with one other study that measured long term mental health of teen mothers in South Africa(37) and with similar research in HIC(14). Poor mental health among teenage mothers can impair parenting capacity and reduce labor force participation, worsening economic outcomes(61, 62). Additionally, depressive symptoms are associated with lower health-seeking behavior, which can compound physical health issues for mothers and children (63)."

19. Need to elaborate on the impact of poor socioeconomic outcomes (increased loans, food insecurity) amongst this group and possible interplay with teenage pregnancy or socioeconomic status and what this could mean?

Response: Great comment, thank you for this. We have added a paragraph that focuses on socioeconomic outcomes and possible interplay with teenage pregnancy and its implications. We believe this significantly strengthens the paper. (pg 13-14).

"Overall, teenage mothers, especially those who had multiple children, face substantially worse socio-economic outcomes as adults than women who first gave birth in adulthood. These outcomes likely reflect both causes and consequences of early childbearing. Low socioeconomic status is a known risk factor for teenage pregnancy, as impoverished girls often lack access to quality education and reproductive health services (21, 64). Becoming a mother during adolescence can exacerbate these hardships, especially when girls drop out of school, which reduces future earning potential and increases the need for informal borrowing or high-interest loans (12, 65, 66). Limited earning capacity also raises the risk of food insecurity, which further harms maternal and child health outcomes (67-69). These adverse socioeconomic conditions trap mothers in debt cycles by reducing their financial independence, (65) limiting access to healthcare (which increases maternal complications, poor birth outcomes, and child mortality),(70) and exacerbating psychological distress such as feelings of hopelessness, depression and anxiety, (which further deepens financial insecurity). Children of teenage mothers who grow up in poverty are also more likely to experience poor health, drop out of school, need income assistance, and repeat cycles of early pregnancy and poverty (12, 71, 72)."

20. 'We had sufficient sample size to explore the topic of teenage pregnancy with greater nuance by dividing the teen mothers into two groups and finding the differential effect of having multiple children as teenager.'

    The grammar of this sentence needs to be fixed. Sample size information and adequacy should be in the methodology.

Response: Thank you for this observation. We have corrected the grammar to improve clarity in the discussion section. We believe this information needs to remain in the discussion since we are expanding on the strength of our data & ability to draw robust conclusions. We also included information about sample size in the methods section (pg. 6) and state the sample size of each study group in the opening of the results (pg. 8). The revised sentence now reads (pg. 14):

"Our larger sample allowed for more nuanced analyses by dividing teen mothers into two groups based on parity to assess the differential effect of multiple adolescent births."

21. Is it possible to elaborate upon and contextualize the recommendations based on the findings and local need/possibility in Kenya.

Response: Thank you, this is a great suggestion. We have revised the Discussion to better contextualize our recommendations within Kenya's current policy and programmatic landscape. We now reference key

national, county, and Lwala policies and interventions that contextualize the recommendations and emphasize the importance of ensuring that these programs intentionally include adolescent mothers. The revised text now reads (pg. 14-15):

> "Addressing teenage pregnancy in western Kenya requires targeted, multisectoral interventions that address both its root causes and long-term consequences. Programs integrating health interventions with financial literacy, economic opportunity, and social support aimed at reducing borrowing and improving long-term economic stability, may be particularly effective. Kenya is already prioritizing investments in the youth population with national initiatives such as the Kenya Youth Employment and Opportunities Project (73) the Return to School policy(74), and the Adolescent Sexual and Reproductive Health Policy. These programs align with Kenya's national priorities to eliminate teenage pregnancy by 2030(75) and empower women (76). At the county level, Migori has identified teen pregnancy as a policy priority in its Multi-Sectoral Action Plan for Adolescents and Youth, encouraging collaboration across health, education, and youth sectors (77). The Lwala Community Alliance also runs programs to improve the economic wellbeing of youth affected by teen pregnancy, including Broadened Horizon, which empowers teen mothers to return to school(40). Our study also underlines the urgent need to address the mental toll of teen pregnancy. The persistent disadvantages young mothers face, including limited education, economic opportunity, food security, and child loss, take a tremendous mental health toll that extends into adulthood. Preventing teenage pregnancy and supporting those who become mothers as adolescents is both a public health emergency and an economic development priority for Kenya, and is essential to interrupting cycles of poverty and inequality. Our findings emphasize the critical need for coordinated multisectoral action to promote long term wellbeing of teenage mothers in Kenya."

----------------------------- Reviewer 2 comments -----------------------------

Reviewer #2: I commend the authors for a strong research study answering an important question based on available data, building on prior research study findings and adding important additional insights to the work and field. The study is well conceived, well written, and well presented.

My single critique is that the abstract says " Our study highlights that teenage motherhood has negative consequences on health and economic functioning that persist until adulthood" when, of course, the authors are well aware that the consequences cannot be so clearly defined and that these are associations (the authors go to great extent in the discussion to be clear about this so I assume this is a typo/missed detail and recommend it is corrected with something along the lines of

"Our study highlights…teenage motherhood IS ASSOCIATED WITH negative consequences…" I have no other specific critiques and recommend the study is accepted with the minor aforementioned change.

Response: Thank you very much for your positive feedback about the paper. We appreciate the constructive comment about the need to remove causal language from the abstract. We changed the sentences where we previously mentioned "consequences" and now state that we are describing an association. The sentence now states: (page 2)

> "Our study highlights that teenage motherhood is associated with worse health and economic functioning that persists into adulthood."
